# Crystal structure and Hirshfeld surface analysis of 2-(4-chloro­phen­yl)-4-(di­meth­oxy­meth­yl)-5-phenyl-1,3-thia­zole

**DOI:** 10.1107/S2056989022005564

**Published:** 2022-05-27

**Authors:** Firudin I. Guseinov, Konstantin I. Kobrakov, Elena V. Shuvalova, Egor I. Tuzharov, Mehmet Akkurt, Sema Öztürk Yıldırım, Ajaya Bhattarai

**Affiliations:** a Kosygin State University of Russia, 117997 Moscow, Russian Federation; b N. D. Zelinsky Institute of Organic Chemistry, Russian Academy of Sciences, 119991 Moscow, Russian Federation; c Department of Physics, Faculty of Sciences, Erciyes University, 38039 Kayseri, Turkey; d Department of Physics, Faculty of Science, Eskisehir Technical University, Yunus Emre Campus 26470 Eskisehir, Turkey; e Department of Physics, Faculty of Science, Erciyes University, 38039 Kayseri, Turkey; f Department of Chemistry, M.M.A.M.C (Tribhuvan University), Biratnagar, Nepal; University of Aberdeen, Scotland

**Keywords:** crystal structure, thia­zole ring, chloro­phenyl ring, C—H⋯π inter­actions, Hirshfeld surface analysis

## Abstract

In the title compound, C_18_H_16_ClNO_2_S, the thia­zole ring subtends dihedral angles of 13.12 (14) and 43.79 (14) ° with the attached chloro­phenyl and phenyl rings, respectively. In the crystal, C—H⋯π inter­actions link the mol­ecules, forming a three-dimensional network. The roles of the various inter­molecular inter­actions were clarified by Hirshfeld surface analysis, which reveals that the most important contributions to the crystal packing are from H⋯H (39.2%), H⋯C/C⋯H (25.2%), Cl⋯H/H⋯Cl (11.4%) and O⋯H/H⋯O (8.0%) contacts.

## Abstract 2

In the title compound, the dihedral angles between the thia­zole ring and its attached chloro­phenyl and phenyl rings are 13.12 (14) and 43.79 (14)°, respectively.

## 1. Chemical context

Thia­zole and its derivatives have attracted much synthetic inter­est due to their anti­microbial, anti­viral, anti-diabetic, diuretic, anti­convulsant, anti­oxidant, anti-HIV, analgesic, anti-inflammatory, neuroprotective and anti­tumor activities (Dondoni 2010 ▸; Grover & Jachak 2015 ▸). In fact, the thia­zole moiety is a prominent structural feature in a variety of natural products, such as vitamin B and penicillin (Yariv *et al.*, 2015 ▸). On the other hand, the thia­zole synthon is also useful in coordination chemistry and catalytic transformations due to its coordination ability and non-covalent bond donor or acceptor character (Gurbanov *et al.*, 2020 ▸). As part of our studies in this area, we now report the synthesis and structure of the title compound and qu­antify its inter­molecular non-covalent inter­actions by Hirshfeld surface analysis. 
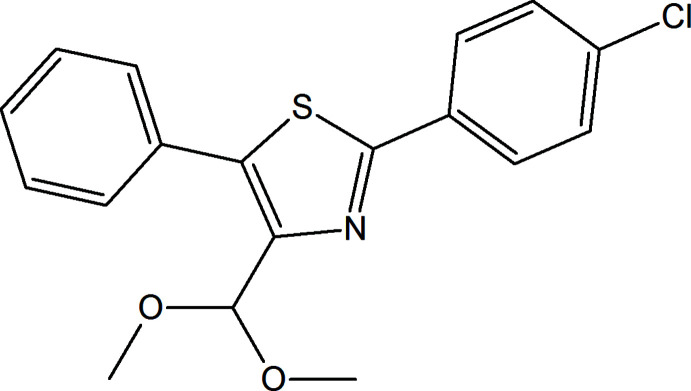


## 2. Structural commentary

**Figure 1:**
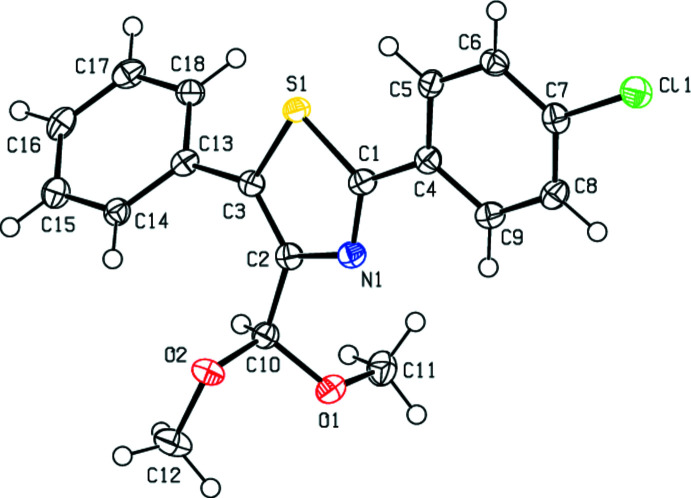
The title mol­ecule with displacement ellipsoids drawn at the 50% probability level.

The mol­ecular structure of the title compound is shown in Fig. 1 ▸. The central 1,3-thia­zolidine ring (S1/N1C1–C3) makes dihedral angles of 13.12 (14) and 43.79 (14)°, respectively, with the chloro­phenyl ring (C4–C9) and the phenyl ring (C13–C18). The di­meth­oxy­methane moiety features one *anti* conformation [C2—C10—O2—C12 = 172.5 (2)°] and one *gauche* conformation [C2—C10—O1—C11 = −78.1 (3)°] for its pendant bonds. The mol­ecular conformation may be consolidated by a weak intra­molecular C5—H5⋯S1 contact [H5⋯S1 = 2.74 Å; C5—H5⋯S1 = 106°].

## 3. Supra­molecular features and Hirshfeld surface analysis

**Figure 2:**
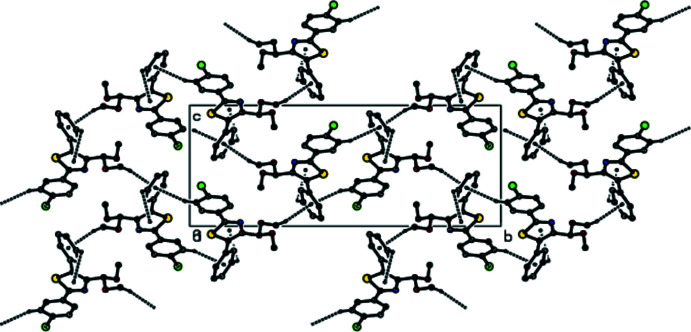
The packing viewed along the *a*-axis direction with the C—H⋯π inter­actions indicated by dashed lines.

**Figure 3:**
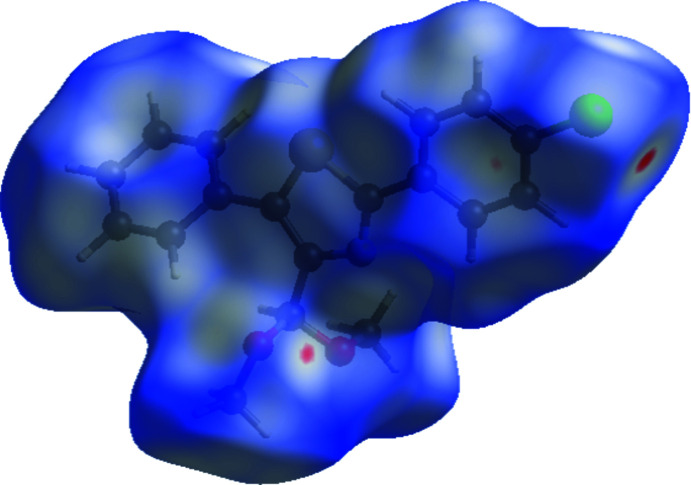
The three-dimensional Hirshfeld surface for the title compound, plotted over *d*_norm_ in the range −0.08 to +1.30 a.u.

**Figure 4:**
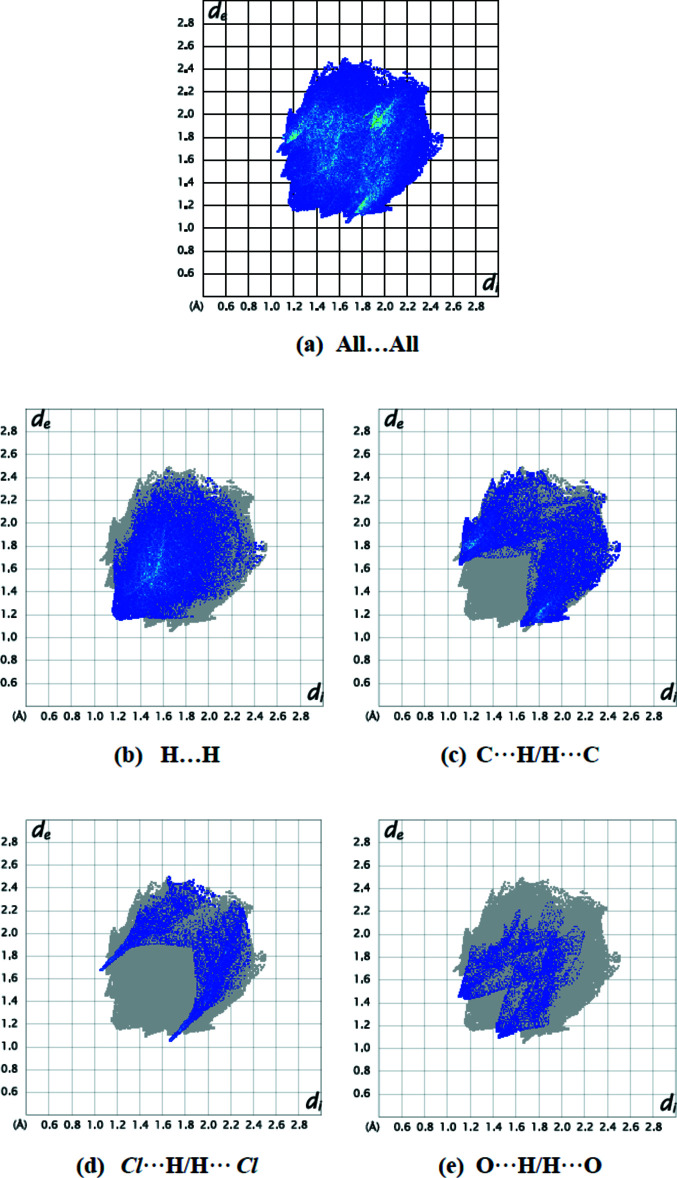
A view of the two-dimensional fingerprint plots for the title compound, showing (*a*) all inter­actions, and delineated into (*b*) H⋯H, (*c*) C⋯H/H⋯C, (*d*) Cl⋯H/H⋯Cl and (*e*) O⋯H/H⋯O inter­actions. The *d*_i_ and *d*_e_ values are the closest inter­nal and external distances (in Å) from given points on the Hirshfeld surface.

The extended structure features C—H⋯π inter­actions, forming a three-dimensional network (Table 1 ▸, Fig. 2 ▸) in which the thia­zole ring accepts once such bond and the phenyl ring two, but no significant π–π stacking contacts are observed [shortest centroid–centroid separation = 4.1887 (16) Å]. A Hirshfeld surface analysis was performed, and two-dimensional fingerprint plots were created with *Crystal Explorer17.5* (Turner *et al.*, 2017 ▸) to qu­antify the inter­molecular inter­actions present in the extended structure. Fig. 3 ▸ depicts the Hirshfeld surface projected on *d*_norm_ and the related colours reflecting various inter­actions. The C—H⋯Cl inter­action is represented by the red spot on the surface. Fig. 4 ▸ depicts the two-dimensional fingerprint plots. The weak van der Waals H⋯H connections provide the most (39.2%, Fig. 4 ▸ *b*) to the Hirshfeld surface. The other principal contributions to the overall surface are from C⋯H/H⋯C (25.2%, Fig. 4 ▸ *c*), Cl⋯H/H⋯Cl (11.4%, Fig. 4 ▸ *d*) and O⋯H/H⋯O (8.0%, Fig. 4 ▸ *e*) inter­actions. The contributions of the remaining less important inter­actions are given in Table 2 ▸.

## 4. Database survey

The most closely related four structures containing the 1,3-thia­zole moiety are as follows: meth­yl(2-(cyclo­pentyl­idenehydrazono)-4-oxo-3-phenyl-1,3-thia­zolidin-5-yl­idene)acetate [Cambridge Structural Database (Groom *et al.*, 2016 ▸) refcode GUVVAW (I); Akkurt *et al.*, 2015 ▸], 2-(5-methyl-4-phenyl-1,3-thia­zol-2-yl)-1-phenyl­ethanol [EKEZUP (II); Rybakov *et al.*, 2003 ▸], 2-{(*E*)-2-[(2-chloro­phen­yl)methyl­idene]hydrazin-1-yl}-4-phenyl-1,3-thia­zole [WOJKOX (III); Mague *et al.*, 2014 ▸] and 2-[4-(4-meth­oxy­phen­yl)-1,3-thia­zol-2-yl]-2,3-di­hydro-1*H*-iso­indole-1,3-dione [IQUHOT (IV); Saravanan *et al.*, 2016 ▸].

In the crystal of (I), the thia­zolidinyl ring (r.m.s. deviation = 0.024 Å) forms a dihedral angle of 65.13 (8)° with the attached phenyl ring. The mol­ecular packing features C—H⋯O and C—H⋯π inter­actions, forming a three-dimensional network. In (II), mol­ecules form extended chains through O—H⋯N hydrogen bonds and in (III), the two independent mol­ecules are associated *via* complementary N—H⋯N hydrogen bonds into a dimer. These dimers are associated through weak C—H⋯Cl and C—H⋯S inter­actions into supra­molecular chains propagating along the *a*-axis direction. In (IV), the mol­ecules are linked *via* C—H⋯O inter­actions, which form *C*(7) chains propagating along [010]. In addition to this, weak π–π inter­actions are also observed.

## 5. Synthesis and crystallization

A mixture of 1-chloro-3,3-dieth­oxy-1-phenyl­propan-2-one (0.769 g, 2 mmol) and 4-chloro­benzo­thio­amide (0.514 g, 3 mmol) was refluxed in methanol (15 ml) for 3 h. Then, the solvent was distilled off in a rotary evaporator under a vacuum. The residue was recrystallized from diethyl ether. Crystals of the title compound suitable for X-ray analysis were obtained by slow evaporation of a acetone solution. Colourless solid, yield 0.891 g (86%); m.p. 401–402 K. Analysis calculated for C_18_H_16_ClNO_2_S: C 62.51, H 4.66, N 4.05; found: C 62.47, H 4.61, N 4.01%. ^1^H NMR (300 MHz, CDCl_3_) *δ* 3.52 (6H, 2CH_3_), 4.62 (1H, CH), 7.22–8.90 (9H, Ar). ^13^C NMR (75 MHz, CDCl_3_) *δ* 169.6, 168.2, 154.4, 144.00, 142.4, 130.8, 129.6, 128.2, 127.4, 126.8, 126.00, 115.2 and 55.8. ESI–MS: *m*/*z*: 346.88 [*M* + H]^+^.

## 6. Refinement details

Crystal data, data collection and structure refinement details are summarized in Table 3 ▸. All H atoms bonded to C atoms were positioned geometrically (C—H = 0.93–1.00 Å) and constrained to ride on their parent atoms with *U*_iso_(H) = 1.2–1.5*U*_eq_(C)

## Supplementary Material

Crystal structure: contains datablock(s) I. DOI: 10.1107/S2056989022005564/hb8023sup1.cif

Structure factors: contains datablock(s) I. DOI: 10.1107/S2056989022005564/hb8023Isup2.hkl

Supporting information file. DOI: 10.1107/S2056989022005564/hb8023Isup3.cml

CCDC reference: 2174374

Additional supporting information:  crystallographic information; 3D view; checkCIF report

## Figures and Tables

**Table 1:** Hydrogen-bond geometry (Å, °) *Cg*1 and *Cg*3 are the centroids of the C1–C3/S1/N1 and C13–C18 rings, respectively.

*D*—H⋯*A*	*D*—H	H⋯*A*	*D*⋯*A*	*D*—H⋯*A*
C5—H5⋯S1	0.95	2.74	3.143 (3)	106
C6—H6⋯*Cg*3^i^	0.95	2.81	3.620 (3)	144
C12—H12*C*⋯*Cg*3^ii^	0.98	2.81	3.406 (3)	120
C15—H15⋯*Cg*1^iii^	0.95	2.95	3.481 (3)	117

Symmetry codes: (i) 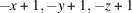 ; (ii) 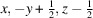 ; (iii) 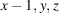 .

**Table 2:** Percentage contributions of inter­atomic contacts to the Hirshfeld surface for the title compound

Contact	Percentage contribution
H⋯H	39.2
H⋯C/C⋯H	25.2
Cl⋯H/H⋯Cl	11.4
O⋯H/H⋯O	8.0
S⋯H/H⋯S	5.1
N⋯H/H⋯N	3.9
C⋯C	2.4
Cl⋯C/C⋯Cl	1.7
S⋯C/C⋯S	1.5
Cl⋯Cl	0.6
S⋯S	0.2
O⋯C/C⋯O	0.1

**Table 3:** Experimental details

Crystal data	
Chemical formula	C_18_H_16_ClNO_2_S
*M*_r_	345.83
Crystal system, space group	Monoclinic, *P*2_1_/*c*
Temperature (K)	100
*a*, *b*, *c* (Å)	6.6235 (1), 25.1848 (3), 9.8283 (1)
β (°)	96.504 (1)
*V* (Å^3^)	1628.92 (4)
*Z*	4
Radiation type	Cu *K*α
μ (mm^−1^)	3.34
Crystal size (mm)	0.2 × 0.12 × 0.04

Data collection	
Diffractometer	XtaLAB Synergy, Dualflex, HyPix
Absorption correction	Multi-scan (*CrysAlis PRO*; Rigaku OD, 2022 ▸)
*T*_min_, *T*_max_	0.638, 1.000
No. of measured, independent and observed [*I* > 2σ(*I*)] reflections	31880, 3497, 3304
*R*_int_	0.064
(sin θ/λ)_max_ (Å^−1^)	0.638

Refinement	
*R*[*F* ^2^ > 2σ(*F* ^2^)], *wR*(*F* ^2^), *S*	0.055, 0.153, 1.12
No. of reflections	3497
No. of parameters	210
H-atom treatment	H-atom parameters constrained
Δρ_max_, Δρ_min_ (e Å^−3^)	0.67, −0.52

Computer programs: *CrysAlis PRO* (Rigaku OD, 2022 ▸), *SHELXT2016/6* (Sheldrick, 2015*a* ▸), *SHELXL2016/6* (Sheldrick, 2015*b* ▸), *ORTEP-3 for Windows* (Farrugia, 2012 ▸) and *PLATON* (Spek, 2020 ▸).

## supplementary crystallographic information

### Crystal data

**Table:**

C_18_H_16_ClNO_2_S	*F*(000) = 720
*M**_r_* = 345.83	*D*_x_ = 1.410 Mg m^−^^3^
Monoclinic, *P*2_1_/*c*	Cu *K*α radiation, λ = 1.54184 Å
*a* = 6.6235 (1) Å	Cell parameters from 20657 reflections
*b* = 25.1848 (3) Å	θ = 3.5–79.0°
*c* = 9.8283 (1) Å	µ = 3.34 mm^−^^1^
β = 96.504 (1)°	*T* = 100 K
*V* = 1628.92 (4) Å^3^	Block, colourless
*Z* = 4	0.2 × 0.12 × 0.04 mm

### Data collection

**Table:**

XtaLAB Synergy, Dualflex, HyPix diffractometer	3497 independent reflections
Radiation source: micro-focus sealed X-ray tube, PhotonJet (Cu) X-ray Source	3304 reflections with *I* > 2σ(*I*)
Mirror monochromator	*R*_int_ = 0.064
Detector resolution: 10.0000 pixels mm^-1^	θ_max_ = 79.5°, θ_min_ = 3.5°
ω scans	*h* = −7→8
Absorption correction: multi-scan (CrysAlisPro; Rigaku OD, 2022)	*k* = −32→32
*T*_min_ = 0.638, *T*_max_ = 1.000	*l* = −12→12
31880 measured reflections	

### Refinement

**Table:**

Refinement on *F*^2^	Primary atom site location: structure-invariant direct methods
Least-squares matrix: full	Secondary atom site location: difference Fourier map
*R*[*F*^2^ > 2σ(*F*^2^)] = 0.055	Hydrogen site location: inferred from neighbouring sites
*wR*(*F*^2^) = 0.153	H-atom parameters constrained
*S* = 1.12	*w* = 1/[σ^2^(*F*_o_^2^) + (0.0621*P*)^2^ + 4.0625*P*] where *P* = (*F*_o_^2^ + 2*F*_c_^2^)/3
3497 reflections	(Δ/σ)_max_ = 0.001
210 parameters	Δρ_max_ = 0.67 e Å^−^^3^
0 restraints	Δρ_min_ = −0.52 e Å^−^^3^

### Special details

**Table:**

Geometry. All esds (except the esd in the dihedral angle between two l.s. planes) are estimated using the full covariance matrix. The cell esds are taken into account individually in the estimation of esds in distances, angles and torsion angles; correlations between esds in cell parameters are only used when they are defined by crystal symmetry. An approximate (isotropic) treatment of cell esds is used for estimating esds involving l.s. planes.
Refinement. Refinement of F^2^ against ALL reflections. The weighted R-factor wR and goodness of fit S are based on F^2^, conventional R-factors R are based on F, with F set to zero for negative F^2^. The threshold expression of F^2^ > 2sigma(F^2^) is used only for calculating R-factors(gt) etc. and is not relevant to the choice of reflections for refinement. R-factors based on F^2^ are statistically about twice as large as those based on F, and R- factors based on ALL data will be even larger.

### Fractional atomic coordinates and isotropic or equivalent isotropic displacement parameters (Å^2^)

**Table:**

	*x*	*y*	*z*	*U*_iso_*/*U*_eq_	
Cl1	1.46338 (11)	0.46239 (3)	0.83382 (7)	0.0279 (2)	
S1	0.51773 (10)	0.42961 (2)	0.43335 (7)	0.01922 (18)	
O1	0.4944 (3)	0.23523 (8)	0.4398 (2)	0.0232 (4)	
O2	0.2192 (3)	0.27028 (8)	0.5252 (2)	0.0234 (4)	
N1	0.6210 (4)	0.33759 (9)	0.5297 (2)	0.0195 (5)	
C1	0.6779 (4)	0.38728 (11)	0.5353 (3)	0.0197 (5)	
C2	0.4449 (4)	0.33113 (11)	0.4441 (3)	0.0194 (5)	
C3	0.3640 (4)	0.37626 (10)	0.3799 (3)	0.0184 (5)	
C4	0.8675 (4)	0.40656 (11)	0.6128 (3)	0.0195 (5)	
C5	0.9086 (4)	0.46059 (11)	0.6290 (3)	0.0204 (5)	
H5	0.809694	0.485762	0.593256	0.025*	
C6	1.0909 (4)	0.47816 (11)	0.6960 (3)	0.0210 (5)	
H6	1.118224	0.515046	0.706735	0.025*	
C7	1.2333 (4)	0.44064 (12)	0.7474 (3)	0.0213 (6)	
C8	1.1971 (4)	0.38669 (12)	0.7341 (3)	0.0229 (6)	
H8	1.296580	0.361728	0.770269	0.028*	
C9	1.0129 (4)	0.36970 (11)	0.6669 (3)	0.0217 (6)	
H9	0.985437	0.332781	0.657512	0.026*	
C10	0.3501 (4)	0.27646 (11)	0.4245 (3)	0.0194 (5)	
H10	0.270809	0.274179	0.331869	0.023*	
C11	0.5988 (5)	0.22879 (13)	0.3220 (3)	0.0296 (7)	
H11A	0.501099	0.230402	0.239378	0.044*	
H11B	0.667819	0.194318	0.326277	0.044*	
H11C	0.699242	0.257220	0.319221	0.044*	
C12	0.0998 (5)	0.22282 (13)	0.5072 (3)	0.0315 (7)	
H12A	0.051128	0.218272	0.410015	0.047*	
H12B	−0.016708	0.225524	0.560094	0.047*	
H12C	0.183243	0.192197	0.539363	0.047*	
C13	0.1824 (4)	0.38355 (11)	0.2793 (3)	0.0188 (5)	
C14	−0.0001 (4)	0.35859 (11)	0.2967 (3)	0.0201 (5)	
H14	−0.009865	0.337216	0.375383	0.024*	
C15	−0.1681 (5)	0.36470 (12)	0.2001 (3)	0.0239 (6)	
H15	−0.291864	0.347268	0.212459	0.029*	
C16	−0.1560 (5)	0.39635 (12)	0.0848 (3)	0.0248 (6)	
H16	−0.270937	0.400469	0.018481	0.030*	
C17	0.0259 (5)	0.42183 (12)	0.0677 (3)	0.0245 (6)	
H17	0.034629	0.443579	−0.010394	0.029*	
C18	0.1943 (4)	0.41573 (11)	0.1636 (3)	0.0213 (6)	
H18	0.317831	0.433298	0.151148	0.026*	

### Atomic displacement parameters (Å^2^)

**Table:**

	*U*^11^	*U*^22^	*U*^33^	*U*^12^	*U*^13^	*U*^23^
Cl1	0.0244 (4)	0.0301 (4)	0.0270 (4)	−0.0046 (3)	−0.0063 (3)	0.0036 (3)
S1	0.0205 (3)	0.0162 (3)	0.0206 (3)	0.0005 (2)	0.0007 (2)	0.0009 (2)
O1	0.0250 (10)	0.0203 (10)	0.0236 (10)	0.0031 (8)	−0.0004 (8)	−0.0006 (7)
O2	0.0281 (11)	0.0210 (10)	0.0217 (10)	−0.0055 (8)	0.0051 (8)	0.0013 (8)
N1	0.0219 (12)	0.0199 (11)	0.0168 (10)	0.0006 (9)	0.0024 (9)	0.0009 (8)
C1	0.0238 (14)	0.0192 (13)	0.0169 (12)	0.0028 (10)	0.0056 (10)	0.0010 (10)
C2	0.0216 (14)	0.0203 (13)	0.0167 (12)	0.0004 (10)	0.0044 (10)	−0.0005 (10)
C3	0.0195 (13)	0.0181 (12)	0.0180 (12)	−0.0005 (10)	0.0030 (10)	−0.0006 (9)
C4	0.0210 (13)	0.0216 (13)	0.0165 (12)	0.0016 (10)	0.0044 (10)	−0.0005 (10)
C5	0.0183 (13)	0.0228 (13)	0.0204 (13)	0.0037 (10)	0.0029 (10)	0.0014 (10)
C6	0.0230 (14)	0.0200 (13)	0.0205 (13)	−0.0015 (10)	0.0047 (11)	−0.0005 (10)
C7	0.0204 (13)	0.0278 (14)	0.0161 (12)	−0.0009 (11)	0.0031 (10)	0.0002 (10)
C8	0.0239 (14)	0.0242 (14)	0.0204 (13)	0.0045 (11)	0.0009 (11)	0.0013 (10)
C9	0.0254 (14)	0.0191 (13)	0.0209 (13)	0.0009 (10)	0.0032 (11)	0.0002 (10)
C10	0.0193 (13)	0.0204 (13)	0.0180 (12)	0.0001 (10)	0.0000 (10)	0.0012 (10)
C11	0.0258 (15)	0.0309 (16)	0.0323 (16)	0.0035 (12)	0.0051 (12)	−0.0037 (12)
C12	0.0367 (18)	0.0257 (15)	0.0318 (16)	−0.0109 (13)	0.0028 (13)	0.0037 (12)
C13	0.0220 (14)	0.0183 (12)	0.0160 (12)	0.0025 (10)	0.0014 (10)	−0.0011 (9)
C14	0.0205 (13)	0.0211 (13)	0.0191 (12)	0.0024 (10)	0.0044 (10)	0.0007 (10)
C15	0.0231 (14)	0.0247 (14)	0.0241 (14)	0.0024 (11)	0.0035 (11)	−0.0017 (11)
C16	0.0243 (14)	0.0275 (14)	0.0212 (13)	0.0075 (11)	−0.0031 (11)	−0.0015 (11)
C17	0.0319 (16)	0.0240 (14)	0.0176 (13)	0.0051 (12)	0.0024 (11)	0.0022 (10)
C18	0.0238 (14)	0.0186 (12)	0.0224 (13)	0.0004 (10)	0.0057 (11)	0.0008 (10)

### Geometric parameters (Å, º)

**Table:**

Cl1—C7	1.747 (3)	C8—C9	1.387 (4)
S1—C1	1.740 (3)	C9—H9	0.9500
S1—C3	1.731 (3)	C10—H10	1.0000
O1—C10	1.408 (3)	C11—H11A	0.9800
O1—C11	1.424 (4)	C11—H11B	0.9800
O2—C10	1.396 (3)	C11—H11C	0.9800
O2—C12	1.433 (4)	C12—H12A	0.9800
N1—C1	1.306 (4)	C12—H12B	0.9800
N1—C2	1.368 (4)	C12—H12C	0.9800
C1—C4	1.475 (4)	C13—C14	1.391 (4)
C2—C3	1.379 (4)	C13—C18	1.406 (4)
C2—C10	1.517 (4)	C14—H14	0.9500
C3—C13	1.480 (4)	C14—C15	1.387 (4)
C4—C5	1.393 (4)	C15—H15	0.9500
C4—C9	1.399 (4)	C15—C16	1.395 (4)
C5—H5	0.9500	C16—H16	0.9500
C5—C6	1.381 (4)	C16—C17	1.392 (4)
C6—H6	0.9500	C17—H17	0.9500
C6—C7	1.389 (4)	C17—C18	1.384 (4)
C7—C8	1.383 (4)	C18—H18	0.9500
C8—H8	0.9500		
			
C3—S1—C1	89.91 (13)	O2—C10—C2	107.0 (2)
C10—O1—C11	112.6 (2)	O2—C10—H10	109.6
C10—O2—C12	112.6 (2)	C2—C10—H10	109.6
C1—N1—C2	111.2 (2)	O1—C11—H11A	109.5
N1—C1—S1	114.1 (2)	O1—C11—H11B	109.5
N1—C1—C4	124.2 (3)	O1—C11—H11C	109.5
C4—C1—S1	121.6 (2)	H11A—C11—H11B	109.5
N1—C2—C3	116.3 (2)	H11A—C11—H11C	109.5
N1—C2—C10	119.9 (2)	H11B—C11—H11C	109.5
C3—C2—C10	123.8 (3)	O2—C12—H12A	109.5
C2—C3—S1	108.4 (2)	O2—C12—H12B	109.5
C2—C3—C13	130.7 (3)	O2—C12—H12C	109.5
C13—C3—S1	120.8 (2)	H12A—C12—H12B	109.5
C5—C4—C1	121.6 (3)	H12A—C12—H12C	109.5
C5—C4—C9	119.2 (3)	H12B—C12—H12C	109.5
C9—C4—C1	119.2 (2)	C14—C13—C3	120.9 (2)
C4—C5—H5	119.4	C14—C13—C18	119.3 (3)
C6—C5—C4	121.1 (3)	C18—C13—C3	119.8 (3)
C6—C5—H5	119.4	C13—C14—H14	119.8
C5—C6—H6	120.8	C15—C14—C13	120.4 (3)
C5—C6—C7	118.4 (3)	C15—C14—H14	119.8
C7—C6—H6	120.8	C14—C15—H15	119.9
C6—C7—Cl1	118.8 (2)	C14—C15—C16	120.3 (3)
C8—C7—Cl1	119.1 (2)	C16—C15—H15	119.9
C8—C7—C6	122.1 (3)	C15—C16—H16	120.3
C7—C8—H8	120.6	C17—C16—C15	119.4 (3)
C7—C8—C9	118.8 (3)	C17—C16—H16	120.3
C9—C8—H8	120.6	C16—C17—H17	119.7
C4—C9—H9	119.8	C18—C17—C16	120.6 (3)
C8—C9—C4	120.5 (3)	C18—C17—H17	119.7
C8—C9—H9	119.8	C13—C18—H18	120.0
O1—C10—C2	112.9 (2)	C17—C18—C13	120.0 (3)
O1—C10—H10	109.6	C17—C18—H18	120.0
O2—C10—O1	108.1 (2)		
			
Cl1—C7—C8—C9	179.2 (2)	C3—C2—C10—O1	150.2 (3)
S1—C1—C4—C5	12.2 (4)	C3—C2—C10—O2	−90.9 (3)
S1—C1—C4—C9	−165.3 (2)	C3—C13—C14—C15	−178.5 (3)
S1—C3—C13—C14	−137.1 (2)	C3—C13—C18—C17	178.7 (2)
S1—C3—C13—C18	43.4 (3)	C4—C5—C6—C7	0.0 (4)
N1—C1—C4—C5	−172.0 (3)	C5—C4—C9—C8	−0.9 (4)
N1—C1—C4—C9	10.5 (4)	C5—C6—C7—Cl1	−179.4 (2)
N1—C2—C3—S1	−0.9 (3)	C5—C6—C7—C8	−0.4 (4)
N1—C2—C3—C13	177.8 (3)	C6—C7—C8—C9	0.2 (4)
N1—C2—C10—O1	−29.8 (3)	C7—C8—C9—C4	0.5 (4)
N1—C2—C10—O2	89.1 (3)	C9—C4—C5—C6	0.7 (4)
C1—S1—C3—C2	0.5 (2)	C10—C2—C3—S1	179.0 (2)
C1—S1—C3—C13	−178.4 (2)	C10—C2—C3—C13	−2.2 (5)
C1—N1—C2—C3	1.0 (3)	C11—O1—C10—O2	163.7 (2)
C1—N1—C2—C10	−179.0 (2)	C11—O1—C10—C2	−78.1 (3)
C1—C4—C5—C6	−176.8 (2)	C12—O2—C10—O1	−65.6 (3)
C1—C4—C9—C8	176.6 (2)	C12—O2—C10—C2	172.5 (2)
C2—N1—C1—S1	−0.5 (3)	C13—C14—C15—C16	−0.5 (4)
C2—N1—C1—C4	−176.6 (2)	C14—C13—C18—C17	−0.7 (4)
C2—C3—C13—C14	44.2 (4)	C14—C15—C16—C17	−0.1 (4)
C2—C3—C13—C18	−135.2 (3)	C15—C16—C17—C18	0.4 (4)
C3—S1—C1—N1	0.0 (2)	C16—C17—C18—C13	0.1 (4)
C3—S1—C1—C4	176.2 (2)	C18—C13—C14—C15	1.0 (4)

### Hydrogen-bond geometry (Å, º)

*Cg*1 and *Cg*3 are the centroids of the C1–C3/S1/N1 and C13–C18 rings, respectively.

**Table:**

*D*—H···*A*	*D*—H	H···*A*	*D*···*A*	*D*—H···*A*
C5—H5···S1	0.95	2.74	3.143 (3)	106
C6—H6···*Cg*3^i^	0.95	2.81	3.620 (3)	144
C12—H12*C*···*Cg*3^ii^	0.98	2.81	3.406 (3)	120
C15—H15···*Cg*1^iii^	0.95	2.95	3.481 (3)	117

Symmetry codes: (i) −*x*+1, −*y*+1, −*z*+1; (ii) *x*, −*y*+1/2, *z*−1/2; (iii) *x*−1, *y*, *z*.

### Summary of short interatomic contacts (Å) in the title compound.

**Table:**

Contact	Distance	Symmetry operation
Cl1···H16	2.85	2 + *x*, *y*, 1 + *z*
H18···Cl1	3.00	2 - *x*, 1 - *y*, 1 - *z*
H11*C*···H15	2.50	1 + *x*, *y*, *z*
H6···C17	2.97	1 - *x*, 1 - *y*, 1 - *z*
O2···H11*A*	2.65	*x*, 1/2 - *y*, 1/2 + *z*
C7···H17	2.85	1 + *x*, *y*, 1 + *z*
C11···H8	3.04	-1 + *x*, 1/2 - *y*, -1/2 + *z*
